# Homogeneous and Heterogeneous Photocatalysis for the Treatment of Pharmaceutical Industry Wastewaters: A Review

**DOI:** 10.3390/toxics10090539

**Published:** 2022-09-16

**Authors:** Maria Antonopoulou

**Affiliations:** Department of Environmental Engineering, University of Patras, 30100 Agrinio, Greece; mantonop@upatras.gr; Tel.: +30-2641074114

**Keywords:** pharmaceutical industry wastewaters, AOPs, photo-Fenton process, heterogeneous photocatalysis, hybrid systems

## Abstract

Pharmaceuticals are biologically active compounds used for therapeutical purposes in humans and animals. Pharmaceuticals enter water bodies in various ways and are detected at concentrations of ng L^−1^–μg L^−1^. Their presence in the environment, and especially long-term pollution, can cause toxic effects on the aquatic ecosystems. The pharmaceutical industry is one of the main sources introducing these compounds in aquatic systems through the disposal of untreated or partially treated wastewaters produced during the different procedures in the manufacturing process. Pharmaceutical industry wastewaters contain numerous pharmaceutical compounds and other chemicals and are characterized by high levels of total dissolved solids (TDS), biochemical oxygen demand (BOD) and chemical oxygen demand (COD). The toxic and recalcitrant nature of this type of wastewater hinders conventional biological processes, leading to its ineffective treatment. Consequently, there is an urgent demand for the development and application of more efficient methods for the treatment of pharmaceutical industry wastewaters. In this context, advanced oxidation processes (AOPs) have emerged as promising technologies for the treatment of pharmaceutical industry wastewaters through contaminant removal, toxicity reduction as well as biodegradability improvement. Therefore, a comprehensive literature study was conducted to review the recent published works dealing with the application of heterogeneous and homogeneous photocatalysis for pharmaceutical industry wastewater treatment as well as the advances in the field. The efficiency of the studied AOPs to treat the wastewaters is assessed. Special attention is also devoted to the coupling of these processes with other conventional methods. Simultaneously with their efficiency, the cost estimation of individual and integrated processes is discussed. Finally, the advantages and limitations of the processes, as well as their perspectives, are addressed.

## 1. Introduction

The release of untreated or partially treated industrial wastewaters in the environment is a considerable source of pollution and can cause a variety of adverse effects on the aquatic environment and human health. In general, industrial activities consume considerable amounts of water and simultaneously produce huge volumes of wastewaters characterized by high toxicity as well as high concentrations of biochemical oxygen demand (BOD), chemical oxygen demand (COD), suspended solids (SS) and inorganic and organic pollutants [1,2].

Pharmaceutical industry wastewater is a characteristic kind of industrial wastewater which contains numerous non-biodegradable organic compounds such as drugs, antibiotics, X-ray constant agents, cytostatic agents, analgesics, anti-inflammatories, antiepileptics, antiseptics, blood lipid regulators, antidepressants, steroids, hormones, flame retardants, and other broadly used chemicals at different concentrations, contributing to high values of BOD, COD and total solids [3]. Due to the enormous volume and hazardous nature of wastewater during manufacturing activities, the pharmaceutical industry is classified as a “red category” [4,5]. The wastewater from pharmaceutical units varies in composition and quantity depending on the raw materials and the processes used in the manufacturing processes. The physicochemical characteristics of pharmaceutical industry wastewaters are summarized in Table 1. Hence, it is very difficult to specify a particular and effective treatment system for pharmaceutical industry wastewaters [3,5,6,7].

In addition, the complex composition of pharmaceutical industry wastewaters, and especially recalcitrant compounds, significantly reduces the performance of conventional wastewater treatment processes. Because of the high concentrations and the non-biodegradable nature of many pharmaceuticals, commonly employed biological and chemical treatment methods are often inefficient for their complete removal [8,9]. Consequently, disposal of treated effluents into receiving aquatic systems can lead to contamination with pharmaceuticals that have proven to cause toxic effects to various microorganisms [6].

High concentrations of pharmaceuticals end up in the environment from various pharmaceutical production facilities. Industrial effluents discharged from the manufacturing units are characterized as a major source entering pharmaceutical compounds in aquatic systems [4,5]. The main pathway for pharmaceuticals to enter the environment is through discharges of pharmaceutical industries wastewater to the wastewater treatment plants (WWTP) and then from municipal effluents. It is estimated that approximately half of the pharmaceutical wastewaters produced worldwide are discarded without specific treatment [6].

Side-effects that have been reported due to the presence of pharmaceutical compounds in the environment and its microorganisms are the development of antibiotic resistance, retardation of nitrite oxidation, methanogenesis, reduction in growth rate of microalgae, feminization in fish and alterations in the behavior and migratory patterns of salmon [6].

Therefore, the efficient treatment of pharmaceutical industry wastewaters is a significant demand before their disposal in water streams to avoid serious environmental problems. As the conventional, treatment technologies have been proven to be inefficient, several alternatives for the treatment of industrial wastewaters have been proposed [10,11]. Over the last years, advanced oxidation processes (AOPs) have received considerable attention due to their high versatility and efficiency in water decontamination. Their efficiency is based on the generation of various species and especially highly oxidative hydroxyl radicals (HO^•^). HO^•^ radicals have high redox potential, are non-selective and able to oxidize various non-biodegradable organic pollutants, such as dyes, pesticides, pharmaceuticals, toxins, etc. In addition to oxidation by HO^•^, several other simultaneous reactions can take place, leading to the degradation of organic pollutants present in wastewater. AOPs offer different possible ways for the in situ formation of the reactive species including several methods such as photo-Fenton, semiconductor photocatalysis, UV/H_2_O_2_, sonolysis, wet air oxidation and O_3_-based processes, among others [10,11,12,13].

Among the AOPs, photocatalysis has proved to be one of the most efficient processes for the treatment of industrial wastewaters. Photocatalysis can take place in homogeneous and heterogeneous phases (depending on the catalysts phase) and involve the generation of reactive transient species such as HO^•^, superoxide anions (O_2_^•−^) and hydroperoxyl (HO_2_^•^) radicals, singlet oxygen (^1^O_2_), holes (h^+^) and electrons (e^−^) [1,10,13]. As HO^•^ radicals possess non-selective nature and high oxidation potential, they present high reaction rates with numerous organic contaminants. Based on the existing literature data, transformation of recalcitrant pollutants in more biodegradable molecules and high mineralization percentages to CO_2_, water and mineral acids under specific conditions can be achieved [1,13,14]

Being aware of the above, numerous research articles are focused on the removal of organic and inorganic contaminants by homogeneous and heterogeneous photocatalysis. In recent years the scientific interest has been extended to the application of homogeneous and heterogeneous photocatalysis for pharmaceutical industry wastewater treatment. Therefore, this study provides an overview on the photocatalytic treatment and purification of real pharmaceutical industry wastewater. The data were collected from the Elsevier Scopus Database documents search with article title, abstract, and keywords as follows: pharmaceutical industry wastewaters, photocatalytic treatment, heterogeneous photocatalysis and photo-Fenton, with the document type “article”. The generated literature list was checked manually and only the articles focused on the treatment of real pharmaceutical industry wastewaters using the targeted processes were considered.

Based on the overview, the efficiency of the processes in terms of COD reduction, pharmaceuticals removal, toxicity abatement and biodegradability increase are discussed. In addition, significant parameters that affect the processes as well as their potential applicability in large scale are evaluated. Special attention is also devoted to their combination with other treatment technologies which can lead to the optimum efficiency with lower cost. Finally, the gaps in the literature that should be investigated are highlighted.

## 2. Photocatalysis in Wastewater Treatment: Fundamental Aspects

Photocatalysis is the acceleration of a photochemical transformation by the action of catalyst such as TiO_2_ or Fenton’s reagent [6]. After the discovery of water splitting by photocatalysis from Fujishima and Honda (1972) [15], this process has been extensively studied for environmental applications, including water and wastewater treatment, using various photocatalysts. One of the most used photocatalysts is TiO_2_ in the forms of anatase and rutile. The band gap of TiO_2_ for anatase and rutile phase is ~3.2 eV and ~3.0 eV, respectively. Its wide application is justified by its significant advantages, i.e., low cost, simple synthesis and chemical and photochemical stability [16,17].

The mixture of anatase/rutile (≈70/30) crystal phases, known as Degussa P25, is one of the most efficient commercially available photocatalytic materials that has been investigated extensively. However, with the development of nanotechnology, doping and co-doping of TiO_2_ with metals and non-metals, the immobilization of the catalysts on a suitable support substrate as well as its combination with other materials have been adopted and studied for the removal of organic pollutants. Other photocatalysts that have also been extensively used to remove pollutants from water are indicatively various sulfides, bismuth oxyhalides, graphitic carbon nitride (g-C_3_N_4_), perovskites and composite materials [13,14,16]. However, most of them have not been investigated for the treatment of real pharmaceutical industry wastewater.

The overall process of heterogeneous photocatalysis can be described by the Equations (1)–(7) included in Table 2 [1,13,16]. TiO_2_ is used as a representative photocatalyst to describe the mechanism. The photocatalytic reaction initiates with the irradiation of TiO_2_ with a photon of energy equal to or greater than its band gap width, and the formation of photogenerated electron/hole (e^−^/h^+^) pairs. In aqueous suspensions the produced holes ($h_{\mathrm{VB}}^{+}$) and electrons ($e_{\mathrm{CB}\;}^{-}$), can react with surface HO^−^ groups and O_2_, respectively, leading to the formation of HO^•^ and $O_{2}^{\cdot -}$ radicals. If oxygen is limited, rapid recombination of photoproduced holes and electrons can take place. The HO_2_^•^ radical is formed by the reaction of proton and $O_{2}^{\cdot -}$ radical. Further reactions can produce HO^•^ through the formation of H_2_O_2_. All these species and especially HO^•^ radicals can react with wastewater pollutants leading to their removal/mineralization. The e^−^ and h^+^ can also lead to the reduction and oxidation of molecules adsorbed on the surface of the photocatalytic material [1,13]. The increase or decrease of the reaction rate is often associated with an enhanced or suppressed e^−^/h^+^ recombination, respectively [11].

In general, five steps take place during photocatalysis [11]:

1. Transfer of molecules to the photocatalyst’s surface;
2. Adsorption of molecules on the surface;
3. Activation of the catalyst and decomposition of adsorbed molecules;
4. Desorption of the products;
5. Removal of reaction products from the photocatalyst’s surface.

The factors that can affect the degradation efficiency are the initial organic load of the wastewater, type of irradiation, mass of catalyst, pH, temperature, irradiation intensity, concentration of oxygen, the addition of oxidants and the presence of substances that can scavenge the reactive species. Furthermore, the separation of heterogeneous catalysts from the treated wastewater, mass transfer limitations on the immobilized catalysts and the low quantum yield for HO^•^ radical production can limit the application of the process in real scale [13].

In the photo-Fenton process, HO^•^ and ${\;O}_{2}^{\cdot -}$ are generated during the irradiation of the H_2_O_2_ and Fe^2+^ mixture (Fenton’s reagent) in acid conditions, according to Equations (8)–(11). The addition of oxalic acid to the solution containing Fe (III) leads to the formation of ferrioxalate complexes (ferrioxalate-assisted photo-Fenton process) that under irradiation can produce also oxidative species such as $O_{2}^{\cdot -}$, HO_2_^•^ and HO^•^ radicals (Equations (12)–(19)) [18].

The main advantage of the photo-Fenton and ferrioxalate-assisted photo-Fenton process is the use of sunlight as source energy since iron-organic acid complexes absorb at wavelengths of the visible light spectrum. The addition of oxalic acid to the photo-Fenton system promotes the formation of ferrioxalate complexes, which expand the useful range of the solar spectrum up to 550 nm and can provide cost-effective and environmentally benign treatment [18]. However, some certain challenges that face these processes in wastewater treatment are the low pH values (~3), the formation of iron sludge as well as the high concentrations of Fe^n+^ ions that can enter the water streams after their application [19,20].

## 3. Homogeneous and Heterogeneous Photocatalysis for the Treatment of Pharmaceutical Industry Wastewaters

### 3.1. Homogeneous Photocatalysis for the Treatment of Pharmaceutical Industry Wastewaters

**Figure 1:**
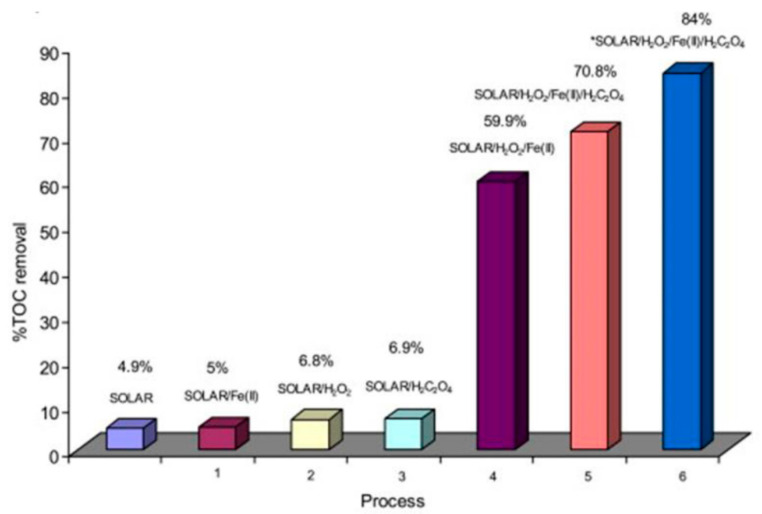
Mineralization of pharmaceutical wastewaters under several systems. (Reaction time: 115 min.; [TOC]_0_ = 125 mg L^−1^; Operating conditions: (1) [Fe(II)]_0_: 55 mg L^−1^; (2) [H_2_O_2_]: 5250 mg L^−1^; (3) [H_2_C_2_O_4_]: 325 mg L^−1^; (4) [H_2_O_2_]: 5250 mg L^−1^; [Fe(II)]_0_: 55 mg L^−1^; (5) [H_2_O_2_]: 5250 mg L^−1^; [Fe(II)]_0_: 55 mg L^−1^; [H_2_C_2_O_4_]: 325 mg L^−1^; (6) [H_2_O_2_]: 5250 mg L^−1^; [Fe(II)]_0_:120 mg L^−1^; [H_2_C_2_O_4_]: 510 mg L^−1^) (Reprinted from [18], with permission of Elsevier 2013).

Homogeneous photocatalytic processes have been studied for the treatment of pharmaceutical industry wastewaters at laboratory and pilot scales [2,7,9,18,21,22,23,24]. Table 3 summarizes the experimental studies applying the homogeneous photo-Fenton and ferrioxalate-assisted photo-Fenton processes for pharmaceutical industry wastewaters treatment. Fenton’s reagent (a mixture of H_2_O_2_ and Fe^2+^) and ferrioxalate complexes in acid pH values under mainly solar irradiation were studied. The removal efficiency was found to depend on the applied experimental conditions as well as the initial organic load of the wastewater. Initial concentrations of Fe (II) and H_2_O_2_ affected the removal efficiency significantly [18]. In case of ferrioxalate-assisted photo-Fenton process, the addition of oxalic acid also enhanced the efficiency [18,23], as depicted in Figure 1 [18]. The increased degradation efficiency could be attributed to the continuous regeneration of Fe (II) via the photo-reduction of Fe (III) and the formation of oxidative species and mainly HO^•^· through ferrioxalate photochemistry [18].

H_2_O_2_ conversion efficiency and the degree of mineralization were the highest when the oxalic/Fe(III) initial molar relation was close to 3. In these conditions, the Fe(III) ions were complexed with the maximum amount of oxalate in the form of the saturated complex Fe(C_2_O_4_)_3_^3−^ [18].

In most of the studies, acidic conditions (≤3) were applied to avoid the precipitation of Fe^3+^ (Table 3). The low pH values needed in photo-Fenton process are one of the main factors that limit its application in wastewater treatment. Iron-based solid catalysts have been investigated the last decades allowing the easy removal of the catalysts from the treated wastewaters. Special attention is also given to magnetically recoverable catalysts and on catalyst immobilization. On the other hand, the possibility of applying solar irradiation in photo-Fenton process reduces the cost of energy consumption, thus providing an important advantage. The photo-Fenton process can be also applied as a pretreatment method to increase the biodegradability of pharmaceutical industry wastewater rendering the biological treatment more efficient. This option is described in Section 3.3.

### 3.2. Heterogeneous Photocatalysis for the Treatment of Pharmaceutical Industry Wastewaters

The published works that have focused on the application of heterogeneous photocatalysis for the treatment of pharmaceutical industry wastewaters are compiled in Table 4. Heterogeneous photocatalysis using commercial TiO_2_ nanoparticles and mainly Degussa P25, Sn-modified TiO_2_ as well as nanocomposites of TiO_2_ with multi-wall carbon nanotubes (MWCNTs) has been studied for the treatment of pharmaceutical industry wastewaters under UV irradiation [25,26,27,28,29,30]. The addition of oxidants, such as H_2_O_2,_ enhanced the photocatalytic removal, probably due to the formation of oxidative species and mainly HO^•^ radicals. Although in most cases high efficiencies in terms of COD and toxicity reduction were achieved, TiO_2_ photoactivity, mainly in the UV region due to the wide bandgap as well as the separation step needed in case of suspensions, limit significantly its application. To overcome these limitations, doping of TiO_2_ and coating of the photocatalyst particles onto supports that are readily removable was investigated and promising results for the treatment of pharmaceutical industry wastewaters were reported [31].

**Figure 2:**
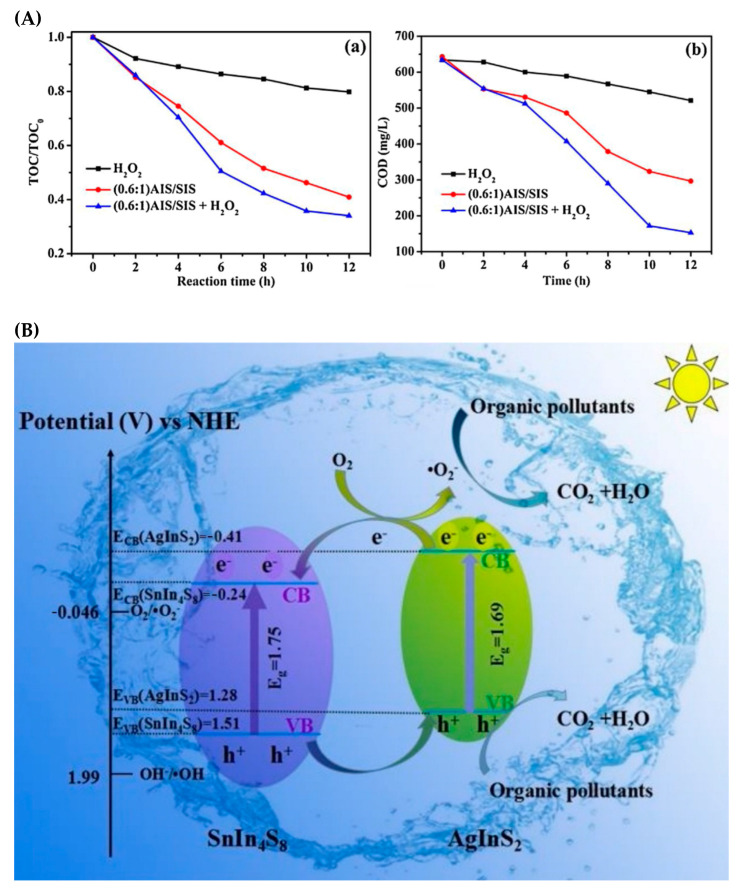
(**A**) The mineralization efficiency of pharmaceutical wastewater (**a**) and the COD removal of real pharmaceutical wastewater by (0.6:1) AIS/SIS heterojunction (**b**). (**B**) Possible photocatalytic mechanism of AIS/SIS heterojunctions (Reprinted from [32], with permission of Elsevier 2017).

As the usage of solar irradiation is essential for large scale applications, visible-light-responsive photocatalysts have been synthesized and investigated for pharmaceutical industry wastewaters treatment. Two-dimensional AgInS_2_/SnIn_4_S_8_ nanosheet heterojunctions with strong visible-light absorption and narrow band gap of 2.27–2.35 eV were prepared and investigated for the treatment of pharmaceutical industry wastewater [32]. About 50% COD removal was observed in 720 min, whereas the addition of H_2_O_2_ enhanced the efficiency (Figure 2A), and the COD of pharmaceutical industry wastewater decreased to 153 mg L^−1^, which meets the discharge standards for industrial effluent. The high photocatalytic activity of AgInS_2_/SnIn_4_S_8_ is attributed to the efficient charge separation (Figure 2B), and its high catalytic stability after five catalytic cycles is correlated with the strong chemical interaction between AgInS_2_ and SnIn_4_S_8_ [32]. In addition, 1% graphene oxide/AgIn5S_8_ (rGO/AgIn5S_8_) nanocomposites were tested for the treatment of real pharmaceutical industry wastewater under visible-light illumination [33]. 76% COD removal was observed in 90 min whereas the addition of H_2_O_2_ led to 89% COD removal in 90 min. The enhanced photocatalytic activity of the composite material was primarily attributed to largest specific surface area, enhanced light absorption as well as the most efficient separation and transfer of photogenerated charge carriers through rGO sheets which act as electron acceptors and transfer channels in the nanocomposites [33].

From an economic point of view, heterogeneous photocatalysis can be applicable for the treatment of pharmaceutical industry wastewaters on a large scale by the usage of solar irradiation. Consequently, much more research is needed, and special efforts should be devoted to design photocatalysts which combine high efficiency, visible-light response, low-cost, stability, good reusability, and environmental friendliness.

### 3.3. Hybrid Systems for the Treatment of Pharmaceutical Industry Wastewaters

Hybrid systems involve several combinations of different technologies for the treatment of industrial wastewaters. Hybrid systems can minimize the disadvantages of individual technologies and simultaneously improve the total efficiency. Photocatalytic processes have been combined with other methods for the treatment of pharmaceutical industry wastewaters and the studied hybrid systems, along with their efficiency, are presented in Table 5.

The combination of heterogeneous photocatalysis and photo-Fenton using Fe-TiO_2_ composite photocatalyst (composite beads) has been applied for the treatment of real pharmaceutical industry wastewaters using various experimental set-ups and irradiation sources [25,34]. Removal of COD higher than 71% was achieved in all cases and the hybrid systems were more efficient than the individual processes [25,34]. A combination of solar photo-Fenton with ozonation has also shown improvement of COD removal compared with individual processes (either solar photo-Fenton or ozonation) [24]. Moreover, the hybrid system led to a significant decrease in operational costs due to the reduction of catalyst consumption, along with the absence of sludge production [24].

Heterogeneous photocatalysis using commercial TiO_2_ nanoparticles as well as WO_3_/CNT under UV and visible irradiation has been combined with sonolysis [26,35] to treat real pharmaceutical wastewaters. In both cases, the combined processes showed higher efficiency than the individual methods in terms of toxicity and COD reduction as well as biodegradability increase [26,35]. Similar results (toxicity and COD reduction and biodegradability increase) were observed when heterogeneous photocatalysis using commercial TiO_2_ nanoparticles was combined with biological treatment (rotating biological contractor (RBC)) to treat real pharmaceutical wastewaters [28].

**Figure 3:**
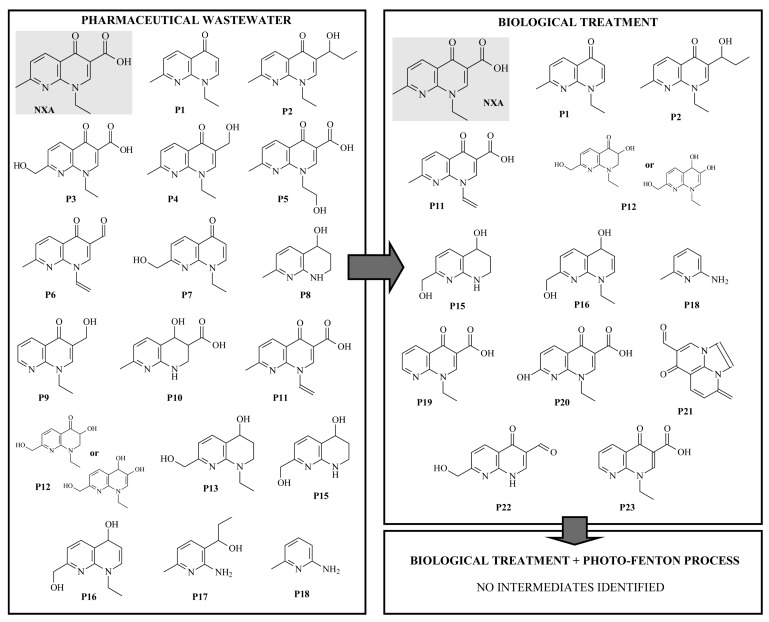
Nalidixic acid and transformation products identified during the treatment of pharmaceutical wastewater (Reprinted from [38], with permission of American Chemical Society, 2009).

Boroski et al. (2009) employed electrocoagulation (EC) followed by UV/TiO_2_/H_2_O_2_ and obtained 97% COD removal [36]. Photo-Fenton as a pre-treatment stage to increase the biodegradability of the wastewater followed by biological process has been investigated by various authors [7,21,22,37]. Overall results indicate that pre-treatment of pharmaceutical wastewater by photo-Fenton with subsequent biological degradation led to higher COD and TOC removal efficiency when compared to individual processes. In addition, complete detoxification of wastewaters was achieved indicating that hybrid treatment technology is an effective approach [7,21,22,37]. Sirtori et al. (2009) [38] investigated solar photo-Fenton as a finishing step for biological treatment of a pharmaceutical wastewater containing mainly nalidixic acid (NXA). Total degradation of NXA and its transformation products after the hybrid process (Figure 3) as well as reduction in toxicity were observed, demonstrating that this hybrid system is a useful and efficient approach [38].

### 3.4. Cost Estimation/Operational Costs

Cost analysis is one of the most significant factors in wastewater treatment [39,40]. For the application of homogeneous and heterogeneous photocatalysis (individually or in combination with other processes) in large-scale, solving simultaneously practical engineering problems, cost estimation must be considered. However, limited studies on cost estimation are available in the literature, fact that renders difficult the large-scale application. An economic analysis was carried out by Monteagudo et al. (2013) using a ferrioxalate-assisted solar photo-Fenton process in a compound parabolic collector (CPC) pilot plant for the treatment of 35 L pharmaceutical wastewater containing 125 mg L^−1^ TOC. The costs considered are related to electrical energy and chemical (reagents and catalysts) consumption. For the maximum mineralization degree, a total cost of EUR 0.0157/g TOC removed or EUR 1.65/m^3^ of treated wastewater was estimated. However, cost reduction could be achieved using more collectors with photovoltaic panels [18].

In a pilot-scale using cascade reactor and solar irradiation, the combination of photocatalysis and photo-Fenton, in situ dual process using Fe-TiO_2_ composite beads, was found to be a good option in terms of cost evaluation (fabrication of reactor, synthesis of composite beads, oxidant, electricity consumption). The overall cost of the treatment process estimated to be USD 0.357 (treatment of 5 L of wastewater) and USD 25.047 for one and 70 runs, respectively. The overall cost of this hybrid system can be further reduced by the reuse of the beads [25] as well as with appropriate engineering modifications and efficient reactor designing in scale-up studies [34]. Based on Talwar et al. (2021), a scale-up cost analysis of the dual process demonstrated that the cost can be less than USD 0.1 L^−1^ of the wastewater [34].

A combination of solar photo-Fenton with ozonation led to a significant decrease in operational costs i.e., chemicals and energy consumption (total cost: EUR 12.69 m^−3^, EUR 1.22 kg^−1^ COD removed) due to the reduction of catalyst consumption, along with the absence of sludge production when used for the treatment of pharmaceutical industry wastewaters [24].

## 4. Conclusions and Prospects for Future Research

The complex composition of pharmaceutical industry wastewaters renders their effective treatment an urgent demand prior to their disposal into the environment. Based on the literature survey conducted in this review, homogeneous and heterogeneous photocatalysis can be characterized as effective methods for the treatment of pharmaceutical industry wastewaters. The main advantages, limitations and prospects of photocatalytic processes for the treatment of pharmaceutical industry wastewaters are summarized in Table 6. In general, high removal percentages can be achieved after the optimization of the processes. However, differences in the efficiency highlighted the importance of performing optimization studies before application. Moreover, the impact of wastewater characteristics on the overall process efficiency was found to be significant. Some limitations of the processes such as continuous usage of chemicals and energy, iron sludge production and separation of the catalyst particles strongly hinder full-scale application, and all these aspects need further investigation. During the last decades, efforts have been made to synthesize materials with visible-light response as alternatives to conventional catalysts. Another aspect of interest is the separation of heterogeneous catalysts and their reuse. As a good solution, magnetic materials in the form of metal oxides, spinels and composites, due to their easy separation by a magnetic field, can be considered. Thus, the application of photocatalytic processes using novel materials should also be extended and investigated for the treatment of pharmaceutical industry wastewaters.

The effect of real wastewater quality on photocatalytic processes is important, and the influence of various matrix components on the efficiency needs to be researched systematically in the future. This can also contribute to the transformation of photocatalytic processes from laboratory into large-/pilot-scale, which is still the challenge. In case of real wastewaters which are produced at flow rates of thousands of m^3^ per day and large-scale applications, the combination of a conventional process with photocatalysis seems to be the most promising option. Hybrid technologies can enhance efficiency, leading to high removal percentages of the pollutants, in most cases within safe discharge limits, as well as to reduce the cost of the treatment process. The most common hybrid treatment scheme that have been studied for pharmaceutical industry wastewaters combines photo-Fenton process, as a pretreatment stage, to remove mainly recalcitrant compounds and enhance the biodegradability of the wastewater and a biological treatment method.

The overall assessment of the literature highlights that special emphasis should be placed on large-scale application for non-biodegradable wastewater treatment and probably reuse. More work is needed to be done on the synthesis and recycling of novel catalysts, matrix impact on degradation kinetics and reactor modeling of the individual and combined processes. A complete economic analysis should also be conducted, including equipment and implementation expenses, amortization, reagent demand, energy costs and sludge disposal. Better economic models must be developed to estimate how the cost of the individual and/or combined processes varies with specific industrial wastewater characteristics as well as the targeted decontamination. Moreover, scarce data exist concerning the identification of the produced transformation products (TPs) of the pharmaceuticals present in the wastewater during the applied processes. Similarly, limited studies evaluated the toxicity evolution during the treatment by the studied AOPs. Considering that undesired intermediates TPs can be formed in some cases, the elucidation of the structures of the TPs, as well as the monitoring of the toxicity evolution, are imperative. These aspects are fundamental for the optimization of the processes and give a useful insight about their potential integration with other process under real conditions as well. The overall future trend is to ensure removal efficiency focusing on the final characteristics of the treated wastewater and the removal of the target contaminants to concentrations safe for the environment while reducing the influence of the other limiting factors.

## Figures and Tables

**Table 1:** Composition of pharmaceutical industry wastewater [3,6,7].

Parameter	Value
pH	3.3–12.7
Color	Light–Dark brown
Conductivity (mS cm^−1^)	1.23–44.85
COD (mg L^−1^)	180–37,410
BOD_5_ (mg L^−1^)	25–21,560
BOD_5_/COD	0.1–0.6
TSS (mg L^−1^)	57–7130
TDS (mg L^−1^)	675–28,000
TKN (mg L^−1^)	165−770
SO_4_^2−^ (mg L^−1^)	160−9000
Cl^−^ (mg L^−1^)	182–6230

**Table 2:** Heterogeneous and homogeneous photocatalysis mechanisms [1,13,16,18].

Heterogeneous Photocatalysis (TiO_2_ Is Used as a Representative Photocatalyst)	
${\mathrm{TiO}}_{2}+{\mathrm{hv}}_{\;}\to {\;\mathrm{TiO}}_{2}\;\left(e_{\mathrm{CB}\;}^{-}+{\;h}_{\mathrm{VB}}^{+}\right)$	(1)
${\mathrm{TiO}}_{2}\left(h_{\mathrm{VB}}^{+}\right)+H_{2}O\to {\mathrm{TiO}}_{2}+H^{+}+{\;\mathrm{HO}}^{\cdot }$	(2)
${\mathrm{TiO}}_{2}\left(h_{\mathrm{VB}}^{+}\right)+{\mathrm{OH}}^{-}\to {\mathrm{TiO}}_{2}+{\mathrm{HO}}^{\cdot }$	(3)
${\mathrm{TiO}}_{2}\;\left(e_{\mathrm{CB}\;}^{-}\right)+{\;O}_{2}\to {\mathrm{TiO}}_{2}+{\;O}_{2}^{\cdot -}$	(4)
${\;O}_{2}^{\cdot -}+H^{+}\to {\mathrm{HO}}_{2}^{\cdot }$	(5)
$2{\mathrm{HO}}_{2}^{\cdot }\to {\;O}_{2}+H_{2}O_{2}$	(6)
$H_{2}O_{2}+O_{2}^{\cdot -}\to {\;\mathrm{HO}}^{\cdot }+{\mathrm{OH}}^{-}+O_{2}$	(7)
Photo-Fenton process	
$\mathrm{Fe}\;\left(\mathrm{III}\right)+{\;H}_{2}O\to \;\mathrm{Fe}\left(\mathrm{III}\right){\left(\mathrm{OH}\right)}^{2+}+H^{+}\;$	(8)
$\mathrm{Fe}\left(\mathrm{III}\right){\left(\mathrm{OH}\right)}^{2+}+\mathrm{hv}\to \mathrm{Fe}\;\left(\mathrm{II}\right)+{\mathrm{HO}}^{\cdot }\;$	(9)
$\mathrm{Fe}\;\left(\mathrm{II}\right)+H_{2}O_{2}\to \;\mathrm{Fe}\;\left(\mathrm{III}\right)+{\mathrm{HO}}^{\cdot }+\mathrm{OH}$ ^−^	(10)
$\mathrm{Fe}\;\left(\mathrm{II}\right)+O_{2}\to \;\mathrm{Fe}\;\left(\mathrm{III}\right)+O_{2}^{\cdot -}$	(11)
Ferrioxalate-Assisted Photo-Fenton Process	
$\mathrm{Fe}\;\left(\mathrm{III}\right){\left[{\left(C_{2}O_{4}\right)}_{3}\right]}^{3-}+\mathrm{hv}\to \mathrm{Fe}\;\left(\mathrm{II}\right)+2C_{2}O_{4}^{2-}+C_{2}O_{4}^{\cdot -}$	(12)
$C_{2}O_{4}^{\cdot -}+O_{2}\to O_{2}^{\cdot -}+2{\mathrm{CO}}_{2}$	(13)
$C_{2}O_{4}^{\cdot -}\to {\mathrm{CO}}_{2}^{\cdot -}+{\mathrm{CO}}_{2}$	(14)
${\mathrm{CO}}_{2}^{\cdot -}+O_{2}\to O_{2}^{\cdot -}+{\mathrm{CO}}_{2}$	(15)
$O_{2}^{\cdot -}+H^{+}\to {\mathrm{HO}}_{2}^{\cdot }$	(16)
${\mathrm{HO}}_{2}^{\cdot }+{\mathrm{HO}}_{2}^{\cdot }\to H_{2}O_{2}+O_{2}\;$	(17)
${\mathrm{CO}}_{2}^{\cdot -}+\mathrm{Fe}\left(\mathrm{III}\right){\left[{\left(C_{2}O_{4}\right)}_{3}\right]}^{3-}\to \mathrm{Fe}\;\left(\mathrm{II}\right)+3C_{2}O_{4}^{2-}+{\mathrm{CO}}_{2}$	(18)
$\mathrm{Fe}\;\left(\mathrm{II}\right)+H_{2}O_{2}+3C_{2}O_{4}^{2-}\to \mathrm{Fe}\left(\mathrm{III}\right){\left[{\left(C_{2}O_{4}\right)}_{3}\right]}^{3-}+{\mathrm{HO}}^{\cdot }+\mathrm{OH}$ ^−^	(19)

**Table 3:** Photo-Fenton and photo-Fenton-assisted processes for the treatment of pharmaceutical industry wastewaters.

Matrix	Characteristics	Catalyst	Reactor/Irradiation Source	Experimental Conditions	Removal Efficiency	Reference
Pharmaceutical industry wastewater (Hyderabad, India)	[TOC]_0_ = 94,420 mg L^−1^ (undiluted)	FeSO_4_•7H_2_O H_2_O_2_ 50% *w*/*v*	UVA-LED strip 6 W (395 nm)	pH = 5; [Fe^2+^]_0_ = 12 g L^−1^; [H_2_O_2_]_0_ = 100 mL L^−1^	46.51% TOC removal in 120 min	[2]
Pharmaceutical industry wastewater (Derabassi, Punjab)	(i) Raw low strength wastewater (LSW) [COD]_0_ = 8370 ± 190 mg L^−1^ [TOC]_0_ = 2350 ± 110 mg L^−1^ (ii) Raw high strength wastewater (HSW) [COD]_0_ = 37,410 ± 225 mg L^−1^ [TOC]_0_ = 8250 ± 145 mg L^−1^	FeSO_4_•7H_2_O H_2_O_2_ 30% *w*/*v*	Glass reactor/Solar irradiation	(i) pH = 3; [Fe^2+^]_0_ = 0.05 mol L^−1^; [H_2_O_2_]_0_ = 0.25 mol L^−1^ (ii) pH = 3; [Fe^2+^]_0_ = 0.1 mol L^−1^; [H_2_O_2_]_0_ = 1 mol L^−1^	(i) 58.4% COD removal in 120 min 26.4% TOC removal in 120 min (ii) 57.1% COD removal in 120 min 31.5% TOC removal in 120 min	[7]
Pharmaceutical industry wastewater (veterinary pharmaceutical industry, Paraíba Valley, São Paulo State)	Fenbendazole and Triclabendazole: main pollutants	FeSO_4_•7H_2_O H_2_O_2_ 30% *w*/*w*	Tubular batch reactor/Low-pressure mercury lamps (254 nm)	pH = 3; [Fe^2+^]_0_ = 2.5 g L^−1^, [H_2_O_2_]_0_ = 30 g L^−1^; Temperature = 20 °C; UV light power = 28 W	74.2 ± 7.2% TOC removal in 60 min	[9]
Pharmaceutical industry wastewater (Castilla-La Mancha)	[TOC]_0_ = 125 mg L^−1^ (after dilution)	FeSO_4_•7H_2_O (COOH)_2_^•^2H_2_O H_2_O_2_ 30% *w*/*v*	Solar-compound parabolic collector (CPC) pilot plant	pH = 2.9; [Fe^2+^]_0_ = 125 mg L^−1^, [H_2_O_2_]_0_ = 5250 mg L^−1^; [H_2_C_2_O_4_]_0_ = 510 mg L^−1^ Temperature = 35.4 °C; Solar power = 38.59 W m^−2^	84% TOC removal in 115 min	[18]
Pharmaceutical industry wastewater	[DOC]_0_ = 775 mg L^−1^ [COD]_0_ = 3420 mg L^−1^ [Nalidixic acid]_0_ = 45 mg L^−1^	FeSO_4_•7H_2_O H_2_O_2_ 30% *w*/*w*	Pilot plant- CPCs/Solar irradiation	pH = 2.6–2.8 [Fe^2+^]_0_ = 20 mg L^−1^; [H_2_O_2_]_0_ = 200–400 mg L^−1^	90% DOC removal in 400 min (180 mM H_2_O_2_ consumed) 100% Nalidixic acid removal in190 min (72 mM H_2_O_2_ consumed)	[21]
Pharmaceutical industry wastewater (Chennai)	[COD]_0_= 5750 mg L^−1^	Fe^2+^/H_2_O_2_	Reactor/solar irradiation UV intensity = 32 ± 2 W m^−2^	pH = 3; [Fe^2+^]_0_ = 1 g L^−1^; [H_2_O_2_]_0_ = 5 g L^−1^	73 % COD removal in 60 min	[22]
Pharmaceutical industry wastewater (pharmaceutical laboratory)	(i) [TOC]_0_ = 274.1 mg L^−1^ (after dilution) (ii) [TOC]_0_ = 20–400 mg L^−1^ (after dilution)	FeSO_4_•7H_2_O (COOH)_2_^•^2H_2_O H_2_O_2_ 30% *w*/*v*	Semi-industrial autonomous solar CPC plant	(i) pH = 2.7; [Fe^2+^]_0_ = 20 mg L^−1^, [H_2_O_2_]_0_ = 2500 mg L^−1^; molar ratio Fe/oxalic acid = 3 (ii) pH = 2.7; H_2_O_2_/Fe^2+^ =20–125; molar ratio Fe/oxalic acid = 3	(i)~60% TOC removal in 300 min (ii) Up to 79% TOC removalin 120 min	[23]
Pharmaceutical industry wastewater	[COD]_0_ = 18,300 mg L^−1^ [TOC]_0_ = 5000 mg L^−1^	FeSO_4_•7H_2_O H_2_O_2_ 50% *w*/*w*	CPC/Solar irradiation	pH = 3; [Fe^2+^]_0_ = 10 mg L^−1^; stoichiometric H_2_O_2_ dose	~30% COD removal in 180 min < 15% TOC removal in 180 min	[24]

**Table 4:** Heterogeneous photocatalysis for the treatment of pharmaceutical industry wastewaters.

Matrix	Characteristics	Catalyst	Reactor/Irradiation Source	Experimental Conditions	Removal Efficiency	Reference
Pharmaceutical industry wastewater (Ambala, Haryana, India). Pre-treated with coagulation (FeCl_3_)	[COD]_0_ = 4800 mg L^−1^	TiO_2_ Degussa P25	Borosilicate glass bowl/UV-A (365 nm)	[Cat.]_0_ = 1 g L^−1^; [H_2_O_2_]_0_ = 300 mg L^−1^	∼75% COD removal in 300 min	[25]
Pharmaceutical industry wastewater	[COD]_0_ = 2500 ± 500 mg L^−1^(diluted)	TiO_2_ Degussa P25	Photochemical Pyrex glass reactor/UV lamps 125 W Average intensity = 25 W m^−2^	[Cat.]_0_ = 1 g L^−1^; [H_2_O_2_]_0_ = 0.075 g L^−1^; pH = 4	90% COD removal in 240 min	[26]
Pharmaceutical industry wastewater (Sfax, Tunisia)	[DOC]_0_ = 170 mg L^−1^ [Ibuprofen]_0_ = 213 mg L^−1^	TiO_2_ Degussa P25	Quartz cylindrical Reactor-UV LEDs 10 W (382 nm)	[Cat.]_0_ = 2.5 g L^−1^; pH = 7.9 Temperature = 25 °C	57% DOC removal in 240 min 100% Ibuprofen removal in 240 min	[27]
Pharmaceutical industry wastewater (Paunta Sahib, Himachal Pradesh, India)	[COD]_0_ = 12,425 mg L^−1^	TiO_2_ Degussa P25	Glass reactor/UV tubes 30 W	[Cat.]_0_ = 0.6 g L^−1^; pH = 3.2	63.7% COD removal in 455 min	[28]
Pharmaceutical industry wastewater (Hyderabad, India)	[TOC]_0_ = 94,420 mg L^−1^	TiO_2_ (rutile phase)	UVA-LED strip 6 W (395 nm)	[Cat.]_0_ = 0.5 g L^−1^; pH = 9 [H_2_O_2_]_0_ = 16 mL L^−1^	4% TOC removal in 840 min (14 h)	[2]
Pharmaceutical industry wastewater (Toluca City, State of Mexico)	[COD]_0_ = 193 mg L^−1^ [TOC]_0_ = 384 mg L^−1^ [Diclofenac]_0_ =104.63 ± 0.05 μg L^−1^ [Ibuprofen]_0_ = 100.40 ± 0.03 μg L^−1^ [Naproxen]_0_ = 1717.31 ± 0.03 μg L^−1^ [Paracetamol]_0_ = 3034.41 ± 0.02 μg L^−1^	Sn-modified TiO_2_	Photochemistry reactor UV lamp 250 Watts (250 nm)	Not reported	73.6% COD removal 94.3% TOC removal 78.8% Diclofenac removal 82.3% Ibuprofen removal 82.7% Naproxen removal 86.9% Paracetamol removal respectively, after the photocatalytic treatment.	[29]
Pharmaceutical industry wastewater	[TOC]_0_ = 1295 mg L^−1^ [COD]_0_ = 2267 mg L^−1^	MWCNT/TiO_2_	Cylindrical quartz photo-reactor -UV 6 W lamps (240 nm)	[Cat.]_0_ = 0.2 g L^−1^; pH = 5	82.4% TOC removal in 240 min 84.9% COD removal in 240 min	[30]
Pharmaceutical industry wastewater	(i) 50% diluted (ii) undiluted	Mg-doped TiO_2_ coated buoyant clay hollow-spheres	Quartz beaker/UV (11 W) Quartz beaker/LED (9 W) Quartz beaker/tungsten light (15 W)	4–5: number of spheres	(i) 72%, 61% and 68% COD removal under LED, UV, and tungsten photon sources in 300 min (ii) 69%, 58% and 66% COD removal under LED, UV, and tungsten photon sources in 300 min	[31]
Pharmaceutical industry wastewater (Jiujiang, China)	[COD]_0_ = 634 mg L^−1^	AgInS_2_/SnIn_4_S_8_ nanosheet (0.6:1 molar ratio)	Visible-light irradiation	(i)[Cat.]_0_ = 200 mg L^−1^; pH = 9 (ii)[Cat.]_0_ = 200 mg L^−1^; pH = 9 [H_2_O_2_]_0_ = 0.5 mL L^−1^	(i) ~50% COD removal in 720 min (12 h) 59.09% mineralization in 720 min (12 h) (ii)78.7% COD removal in 720 min (12 h) 65.98% mineralization in 720 min (12 h)	[32]
Pharmaceutical industry wastewater (Jiangxi Chemedir)	[COD]_0_ = 31,500 mg L^−1^	1% graphene oxide/AgIn_5_S_8_	Double jacketed glass beaker- Xe lamp 300 W with a 400 nm cut-off filter, Intensity= 1.8 W cm^−2^	(i)[Cat.]_0_ = 400 mg L^−1^ (ii)[Cat.]_0_ = 400 mg L^−1^ [H_2_O_2_]_0_ = 10 mL L^−1^	(i)76% COD removal in 90 min (ii) 89% COD removal in 90 min	[33]

**Table 5:** Hybrid systems (coupling of homogeneous/heterogeneous photocatalysis with other processes) for the treatment of pharmaceutical industry wastewaters.

Matrix	Details	Hybrid Process	Removal Efficiency	Reference
Pharmaceutical industry wastewater (Ambala, Haryana, India). Pre-treated with coagulation (FeCl_3_)	[COD]_0_ = 4800 mg L^−1^	Photocatalysis-Photo-Fenton Fe-TiO_2_ composite beads; Catalyst dose equivalent to 102% area of reactor bed covered with Fe-TiO_2_ composite beads; [H_2_O_2_]_0_ = 1155 mg L^−1^ (i) Pilot-scale under natural solar irradiation; Time: 120 min (ii) Batch mode under artificial UV-A irradiation; Time: 360 min	(i) ∼80% COD removal (ii) 89.1% COD removal	[25]
Pharmaceutical industry wastewater (Barnala, Punjab, India)	[COD]_0_ = 1250 mg L^−1^	Photocatalysis-Photo-Fenton Fe-TiO_2_ composite beads; Number of beads =: 98 (98% surface area covered); [H_2_O_2_]_0_ = 800 mg L^−1^ (i) Batch reactor/solar irradiation; Time: 365 min (ii) Continuous recirculation fixed bed reactor/solar irradiation; Time: 300 min	(i) 71% COD removal (ii) 75% COD removal	[34]
Pharmaceutical industry wastewater	[COD]_0_ = 18,300 mg L^−1^ [TOC]_0_ = 5000 mg L^−1^	Solar photo-Fenton-ozonation (SPFO) pH = 3; [Fe^2+^]_0_ = 10 mg L^−1^; O_3_ = 0.1 g min^−1^; stoichiometric H_2_O_2_ dose; Time: 120 min	~60% COD removal ~12% TOC removal	[24]
Pharmaceutical industry wastewater (Tehran, Iran)	[COD]_0_ = 2429 mg L^−1^ [TOC]_0_ = 702 mg L^−1^	Sono-photocatalysis process WO_3_/CNT; photocatalyst, Visible light pH = 9.0, [Cat.]_0_ = 0.7 g L^−1^, US power: 250 W m^−2^; irradiation intensity = W m^−2^ Time: 220 min	90.6 % COD removal 83.7 % TOC removal	[35]
Pharmaceutical industry wastewater	[COD]_0_ = 2500 ± 500 mg L^−1^(diluted)	Ultrasound- UV/TiO_2_/H_2_O_2_ Ultrasound 100 W, 33 ± 3 kHz UV/TiO_2_/H_2_O_2_: pH 4.0 [Cat.]_0_ = 1 g L^−1^; [H_2_O_2_]_0_ = 0.075 g L^−1^ Time: 240 min	99% COD removal	[26]
Pharmaceutical industry wastewater (Parana State, Brazil)	[COD]_0_= 1753 mg L^−1^	Electrocoagulation (EC) -UV/TiO_2_/H_2_O_2_ EC: iron cathode/anode (12.50 cm × 2.50 cm × 0.10 cm), current density 763 A m^−2^, pH= 6.0 Time: 90 min UV/TiO_2_/H_2_O_2_: pH 3.0, Time: 240 min [Cat.]_0_ = 0.255 g L^−1^; [H_2_O_2_]_0_ = 10 mmol L^−1^	97% COD removal	[36]
Pharmaceutical industry wastewater (Kancheepuram District, Tamil Nadu, India)	[COD]_0_= 25,600 mg L^−1^ [BOD_3_]_0_= 4890 mg L^−1^	Solar photo-Fenton-Activated sludge process Solar photo-Fenton: pH = 3; dosage of H_2_O_2_ 65 mL and Fe^2+^ = 1.34 g Activated sludge process: MLSS concentration of 3490 mg L^−1^	95 % COD removal 93 % BOD removal	[37]
Pharmaceutical industry wastewater (Chennai)	[COD]_0_= 5750 mg L^−1^	Solar photo-Fenton-Aerobic sequential batch reactor (SBR) Solar photo-Fenton: pH = 3; [Fe^2+^]_0_ = 1 g L^−1^; [H_2_O_2_]_0_ = 5 g L^−1^; Time: 60 min SBR: [Biomass]= 4000 mg L^−1^; Time: 300 min	98 % COD removal	[22]
Pharmaceutical industry wastewater (Derabassi, Punjab)	(i) Raw low strength wastewater (LSW) [COD]_0_= 8370 ± 190 mg L^−1^ [TOC]_0_= 2350 ± 110 mg L^−1^ (ii) Raw high strength wastewater (HSW) [COD]_0_= 37,410 ± 225 mg L^−1^ [TOC]_0_= 8250 ± 145 mg L^−1^	Solar photo-Fenton-Aerobic biological treatment Solar photo-Fenton: (i) pH = 3; [Fe^2+^]_0_ = 0.05 mol L^−1^; [H_2_O_2_]_0_ = 0.25 mol L^−1^; Time: 120 min (ii) pH = 3; [Fe^2+^]_0_ = 0.1 mol L^−1^; [H_2_O_2_]_0_ = 1 mol L^−1^; Time: 120 min Aerobic biological treatment: pH = 7.0–8.2; MLSS concentration of 3200 ± 110 mg L^−1^	(i) ~84% COD removal (ii) ~82% COD removal	[7]
Pharmaceutical industry wastewater	[DOC]_0_ = 775 mg L^−1^ [COD]_0_ = 3420 mg L^−1^ [Nalidixic acid]_0_ = 45 mg L^−1^	Solar Photo-Fenton process- Immobilized Biomass Reactor—IBR (biological treatment) Solar Photo-Fenton process: Solar irradiation, pH = 2.6–2.8 [Fe^2+^]_0_ = 20 mg L^−1^; [H_2_O_2_]_0_ = 66 mM; Time: 190 min IBR: pH = 7.0, operation flux = 500 L h^−1^, Time= 7200 min (120 h)	95% DOC removal	[21]
Pharmaceutical industry wastewater	[DOC]_0_ = 725 mg L^−1^ [COD]_0_ = 3400 mg L^−1^ [Nalidixic acid]_0_ = 38 mg L^−1^	Immobilized Biomass Reactor—IBR (biological treatment)- Solar Photo-Fenton process IBR: pH = 7.0, operation flux = 500 L h^−1^, Time: 5760 min (96 h) Solar Photo-Fenton process: Solar irradiation, pH = 2.6–2.8 [Fe^2+^]_0_ = 20 mg L^−1^; [H_2_O_2_]_0_ = 200–400 mg L^−1^; Time: 25 min	96% DOC removal 100% removal of Nalidixic acid	[38]
Pharmaceutical industry wastewater (Paunta Sahib, Himachal Pradesh, India)	[COD]_0_ = 12,425 mg L^−1^	UV/TiO_2_-Rotating Biological Contractor (RBC) UV/TiO_2_: pH = 3.20, [Cat.]_0_ = 0.6 g L^−1^, Time= 455 min RBC: Time:50,400 min (35 d)	96.5% COD removal	[28]

**Table 6:** Advantages, limitations, and prospects of photocatalytic processes for the treatment of pharmaceutical industry wastewaters.

Advantages
High oxidation potential of HO^•^ radicals
Applicable in wide range of pH (in case of heterogeneous photocatalysis)
High removal percentages after optimization
Potential of using solar light
High efficiency in hybrid systems
**Limitations**
Iron sludge production and separation of the catalyst particles
Acid conditions for photo-Fenton and photo-Fenton-assisted processes
High cost due to energy consumption and chemicals usage
Efficiency depends on wastewater characteristics
**Prospects**
Combination with conventional methods
Cost reduction using solar energy
Preparation of novel catalysts with response to visible light good reusability
Large-scale application

## Data Availability

Data are contained within the article.
